# Antagonizing the spindle assembly checkpoint silencing enhances paclitaxel and Navitoclax-mediated apoptosis with distinct mechanistic

**DOI:** 10.1038/s41598-021-83743-7

**Published:** 2021-02-18

**Authors:** Ana C. Henriques, Patrícia M. A. Silva, Bruno Sarmento, Hassan Bousbaa

**Affiliations:** 1 CESPU, Instituto de Investigação e Formação Avançada em Ciências e Tecnologias da Saúde, Instituto Universitário de Ciências da Saúde, Rua Central da Gandra 1317, Gandra, 4585-116 Paredes, Portugal; 2 INEB, Instituto Nacional de Engenharia Biomédica, Universidade Do Porto, Porto, Portugal; 3 Centro Interdisciplinar de Investigação Marinha e Ambiental (CIIMAR/CIMAR), Universidade Do Porto, Porto, Portugal; 4 i3S-Instituto de Investigação e Inovação Em Saúde, Universidade Do Porto, Porto, Portugal

**Keywords:** Cancer, Cell biology, Molecular biology, Oncology, Target validation, siRNAs

## Abstract

Antimitotic drugs arrest cells in mitosis through chronic activation of the spindle assembly checkpoint (SAC), leading to cell death. However, drug-treated cancer cells can escape death by undergoing mitotic slippage, due to premature mitotic exit. Therefore, overcoming slippage issue is a promising chemotherapeutic strategy to improve the effectiveness of antimitotics. Here, we antagonized SAC silencing by knocking down the MAD2-binding protein p31^comet^, to delay mitotic slippage, and tracked cancer cells treated with the antimitotic drug paclitaxel, over 3 days live-cell time-lapse analysis. We found that in the absence of p31^comet^, the duration of mitotic block was increased in cells challenged with nanomolar concentrations of paclitaxel, leading to an additive effects in terms of cell death which was predominantly anticipated during the first mitosis. As accumulation of an apoptotic signal was suggested to prevent mitotic slippage, when we challenged p31^comet^-depleted mitotic-arrested cells with the apoptosis potentiator Navitoclax (previously called ABT-263), cell fate was shifted to accelerated post-mitotic death. We conclude that inhibition of SAC silencing is critical for enhancing the lethality of antimitotic drugs as well as that of therapeutic apoptosis-inducing small molecules, with distinct mechanisms. The study highlights the potential of p31^comet^ as a target for antimitotic therapies.

## Introduction

Microtubule targeting agents (MTAs) such as paclitaxel prevent the spindle assembly during mitosis, leading to chronic activation of the spindle assembly checkpoint (SAC), which results in the mitotic arrest of cancer cells until dead^1–3^. However, the efficacy of these drugs is limited by neurotoxic and hematopoietic side effects, as well as by resistance mechanisms^1,3^. Therefore, alternative antimitotic therapies that do not directly interfere with microtubules have led to the development of the second generation antimitotics^3^. As such, SAC-related proteins have been regarded as potential targets for the development of new strategies to kill cancer cells or to increase their sensitivity to the current chemotherapeutics^3^.

SAC is a mitotic surveillance mechanism that prevents metaphase to anaphase transition until all chromosomes are correctly attached to the spindle microtubules and bi-orientated in the metaphase plate^3–5^. Improper chromosome-microtubule attachments that occur during a normal mitosis, or artificially under MTA treatment, activate the SAC. The SAC activity is mediated by the mitotic checkpoint complex (MCC), which is formed between the SAC proteins Mad2 (mitotic arrest deficiency 2), Bub3 (budding uninhibited by benomyl 3) and Bub1-related 1 (BubR1), and Cdc20 (cell-division cycle protein 20). At unattached kinetochores, closed-Mad2 conformation (C-Mad2) bound to Mad1 recruits open-Mad2 conformation (O-Mad2), thereby promoting its conformational change to C-Mad2^6,7^, which in turn sequesters Cdc20, an activator of anaphase promoting complex/cyclosome (APC/C). Then, Cdc20-C-Mad2 binds to BubR1-Bub3 complex, forming the MCC. As a result of Cdc20 sequestration, the APC/C is kept inactive, thereby preventing securin and cyclin B degradation by the 26S proteasome and, thus, mitotic exit^3–5,8^. One all chromosomes are correctly aligned at the metaphase plate, the SAC must be silenced in order for the cell to proceed to anaphase. The Mad2-binding protein p31^comet^^9,10^ is crucial to this SAC silencing, by blocking further Mad2 activation, and promoting MCC disassembly^11,12^.

The fate of mitotic-arrested cells was reported to be dictated by two competing networks^13^. One network determines cell death through accumulation of apoptotic signals during mitosis. The other network determines mitotic slippage through gradual degradation of cyclin B1. The network that reaches its threshold first determines the cell fate. Therefore, theoretically, it should be possible to have a control over the cell fates and influence the effectiveness of antimitotics if mitotic slippage is retarded and/or death signal accumulation is accelerated^3^.

In this context, and given its key role in SAC silencing and mitotic exit, p31^comet^ appears as an ideal target to delay mitotic slippage. On the other hand, the BH3-only proteins, Bim, Bid, Bad and Noxa, have been shown to contribute to death in mitosis^14–17^. Thus, using BH3-mimetic drugs, in a background of mitotic slippage delay, should shift the fate of mitosis-arrested cells in favor of death. Therefore, we tested these two possibilities by monitoring cell fates by single-cell tracking during three day live-cell time-lapse analysis. Firstly, we investigated the relative contribution of delaying mitotic slippage (through p31^comet^ depletion) to cell death following exposure to nanomolar concentrations of paclitaxel. Secondly, we determined the relative contribution of BH3-mimetic-mediated apoptosis potentiation to cell death of cells delayed in mitosis by p31^comet^ depletion.

## Results

### p31^comet^ expression and knockdown

**Figure 1:**
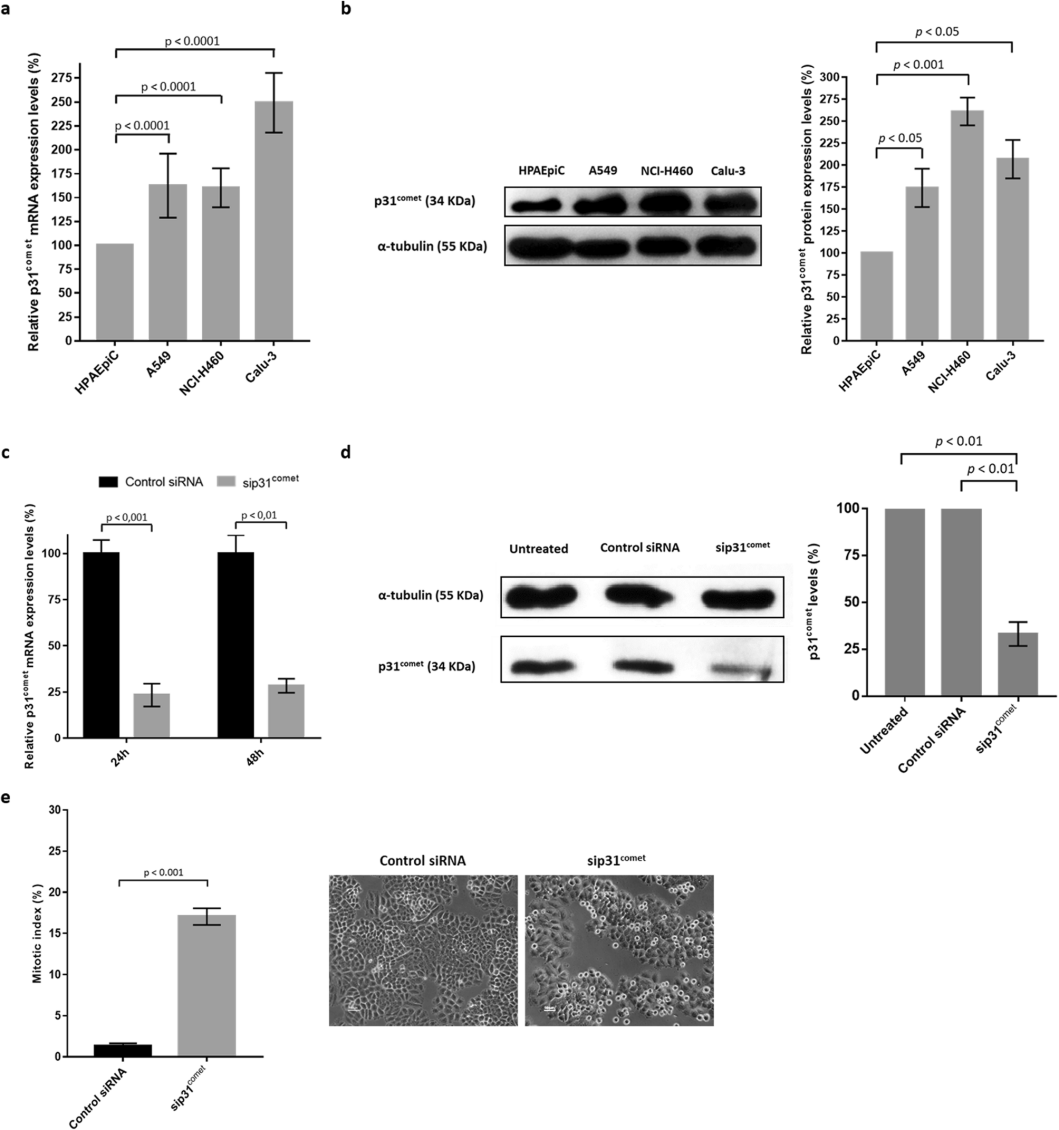
p31^comet^ expression and knockdown in lung cancer cells. p31^comet^ is overexpressed in lung cancer cells, as shown in (**a**, **b**). (**a**) Relative expression of p31^comet^ mRNA as determined by qRT-PCR in the indicated tumor cell lines, comparatively to non-tumor HPAEpiC. (**b**) Representative western blot showing differential expression at protein levels in cell lines analyzed in (**a**) and the respective quantification on the right (full-length blot image in Supplementary Fig. S1). Tubulin served as a loading control. (**c**) Relative expression of p31^comet^ mRNA in control siRNA and in cells treated with p31^comet^ siRNAs (sip31^comet^), as determined by qRT-PCR. RNA was extracted 24 h and 48 h after transfection. (**d**) Representative western blot for NCI-H460 extract showing effective protein depletion (left) and respective quantification (right) (full-length blot image in Supplementary Fig. S2). Tubulin served as a loading control. Protein extraction was performed 48 h after transfection. (**e**) p31^comet^ knockdown increases the mitotic index. Mitotic cells were quantified 48 h after transfection. Statistical analysis was performed through Student t-test. The error bars represent mean ± SD.

In order to obtain a better understanding on the relevance of p31^comet^ as a potential target for cancer therapy, p31^comet^ expression was assessed in three non-small lung cancer cell lines (NSCLC): NCI-H460, A549 and Calu-3, and compared to the non-tumor cell line HPAEpiC. Upregulation of p31^comet^ was observed both at mRNA and protein levels in all the NSCLC cells tested comparatively to HPAEpiC (Fig. 1a,b). This highlights the importance of targeting p31^comet^. Due to its suitability for quantitative evaluation of morphological changes in in vivo microscopy assays, NCI-H460 cell line was selected in this study.

p31^comet^ knockdown was performed using siRNA duplexes previously validated^18^ and ascertained by qRT-PCR and immunoblotting against p31^comet^. More than 70% depletion of p31^comet^ was achieved, both at mRNA and protein levels, 24 h after treatment of NCI-H460 cells with p31^comet^ siRNAs (sip31^comet^), comparatively to cells treated with a negative control siRNA (Control siRNA) (Fig. 1c,d). These depletion levels were not altered by extended transfection time. Furthermore, contrast-phase microscopy analysis revealed an accumulation of round shaped mitotic cells and an increase in the mitotic index (Fig. 1e), in accordance with previously reported p31^comet^ depletion phenotype^19,20^.

### p31^comet^ depletion enhances lethality of nanomolar concentrations of paclitaxel by promoting massive cell death in mitosis

**Figure 2:**
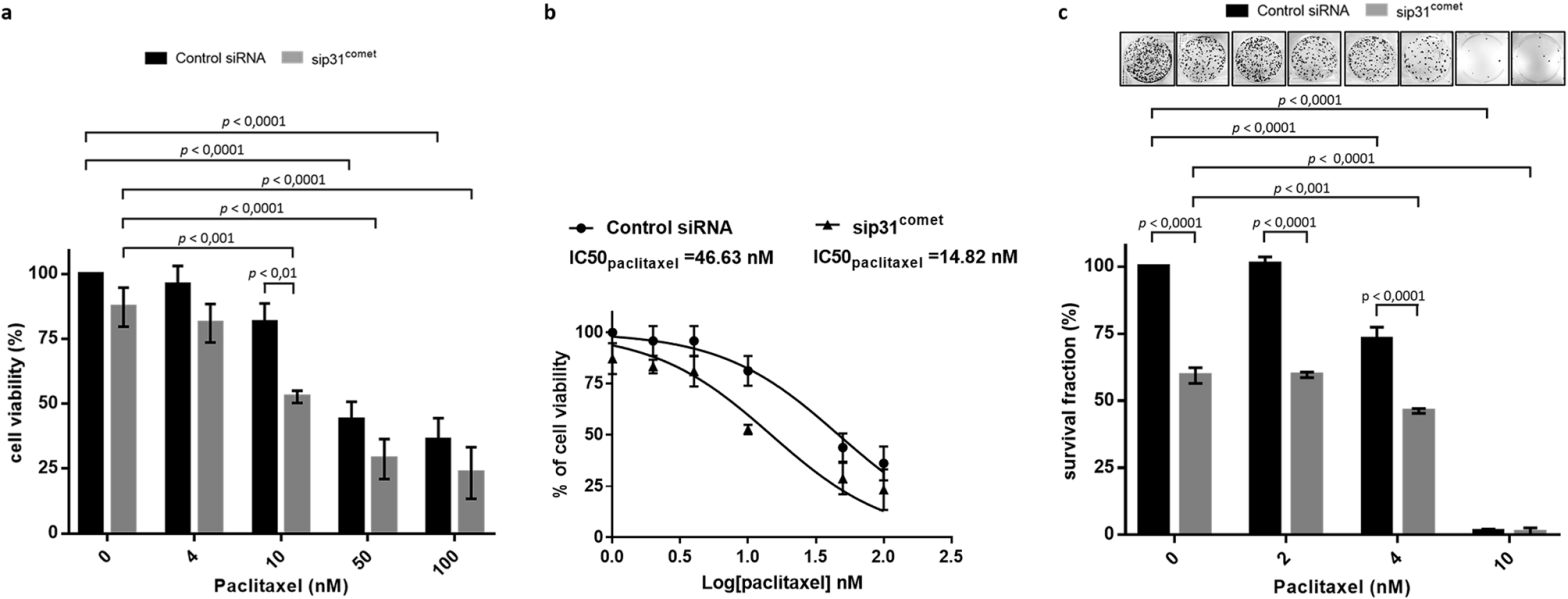
p31^comet^ inhibition enhances paclitaxel-mediated toxicity in NCI-H460 cells under low doses of paclitaxel. (**a**) Cell viability, as determined by MTT assay. Twenty-four hours post-transfection with control or p31^comet^ siRNAs, paclitaxel was added at the indicated concentrations (0–100 nM) and cells were incubated for an extra 48 h. (**b**) Dose response curves and IC50 values for paclitaxel treatment of control or p31^comet^ siRNA-transfected cells. (**c**) Cells were treated as in (**a**), washed and allowed to grow for 10 days for colony formation assays. Results are the mean from three independent experiments, expressed as % of survival fraction. Representative images of surviving colonies (top) are shown for each condition. Statistical analysis was performed by two-way ANOVA with Tukey's multiple comparisons test. The error bars represent mean ± SD.

We explored whether delaying mitotic slippage, by antagonizing SAC silencing through p31^comet^ depletion, could potentiate cancer cell killing to nanomolar concentrations of paclitaxel, ranged from 0 to 100 nM. This is relevant as paclitaxel is used as first line chemotherapy for various cancers^1,21^. We found that paclitaxel concentrations ≥ 50 nM were needed to induce a significant increase in cytotoxicity (*p* < 0.0001), as determined by a 48 h MTT assays (Fig. 2a). In contrast, in cells depleted of p31^comet^, concentrations as low as 10 nM of paclitaxel were sufficient to significantly reduce cell viability (*p* < 0.01). Indeed, dose response curves showed a threefold decrease in IC50 values for paclitaxel in cells depleted of p31^comet^ (Fig. 2b). Importantly, in a 10 days clonogenic assay, p31^comet^ knockdown significantly affected NCI-H460 cell proliferation at much lower concentrations (4 nM) of paclitaxel (*p* < 0.0001), suggesting that long-term survival becomes compromised at very low concentrations of paclitaxel (Fig. 2c). These results indicate that antagonizing SAC silencing by targeting p31^comet^ can enhance lethality of cancer cells in the presence of low doses of paclitaxel. According to the data outlined in (Fig. 2a,b), we selected the concentration of 10 nM paclitaxel to further analyze the mechanism of its combination with p31^comet^ suppression. It represents the lowest concentration that still leads to maximal antitumor effect when combined with p31^comet^ suppression. From a therapeutic point of view, this is expected to reduce paclitaxel toxicity and resistance concerns.

**Figure 3:**
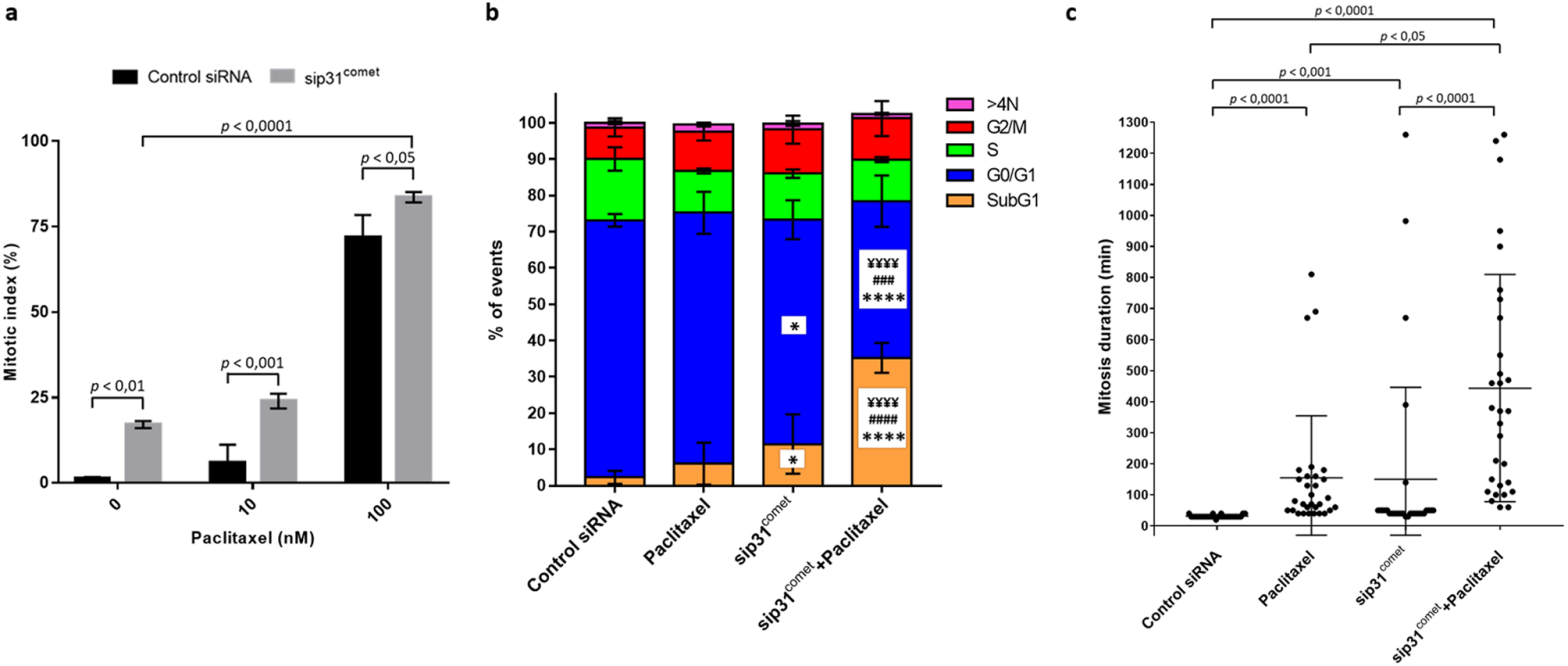
p31^comet^ knockdown increases the mitotic index and the duration of the mitotic block, and enhances cancer cells death under low doses of paclitaxel treatment. NCI-H460 cells were treated with control or p31^comet^ siRNAs for 48 h, then 10 nM of paclitaxel was added for further 24 h or 48 h. (**a**) Determination of the mitotic index 24 h after addition of paclitaxel, as determined phase-contrast microscopy. Two-way ANOVA with Tukey's multiple comparisons test was used for statistical analysis. (**b**) Cell cycle analysis. 48 h after the addition of paclitaxel, cells were treated with propidium iodide/RNase and analyzed by flow cytometry. *p < 0.05, ****p < 0.0001, sip31^comet^ or sip31^comet^ + paclitaxel vs control siRNA; ^###^*p* < 0.001, ^####^*p* < 0.0001, sip31^comet^ + paclitaxel vs sip31^comet^; ^**¥¥¥¥**^p < 0.0001, sip31^comet^ + paclitaxel vs paclitaxel, by two-way ANOVA with Tukey's multiple comparisons test. (**c**) Quantification of mitosis duration. 24 h after paclitaxel treatment and/or 48 h after siRNA transfection cells were followed by time-lapse imaging for 72 h. The scatter plot shows the time from mitosis entry to cell division. Each spot represents one cell. Statistical analysis was performed by Krushkal–Wallis with Dunn’s multiple correction test. The error bars represent mean ± SD.

In order to get insight into the mechanism underlying the lethality enhancement resulting from combining p31^comet^-depletion with clinically relevant concentrations of paclitaxel, we first determined the mitotic index by phase-contrast microscopy and flow cytometry. We observed an increase in the mitotic index in p31^comet^-depleted cells treated with paclitaxel for 24 h (23.92 ± 2.16%), when compared to untreated (1.33 ± 0.31%) and to paclitaxel-treated cells (6.00 ± 5.1%) (Fig. 3a). Flow cytometry analysis not only confirmed these results (Fig. 3b) but also revealed an increased sub-G1 population in p31^comet^-depleted cells treated with 10 nM paclitaxel for 48 h, indicative of massive cell death. This suggests that p31^comet^ depletion is acting as a coadjuvant of paclitaxel, namely at 10 nM, by retaining cells in mitosis through preventing SAC silencing and, thus, delaying mitotic slippage, which may explain the enhancement of cell killing obtained in the above cytotoxic assays.

Then, we scored the duration of mitosis and the survival fate of each mitotic NCI-H460 cell by single-cell time-lapse imaging. For survival fate analysis, we considered three categories: cell death in mitosis (DiM), post-mitotic death (PMD), and survivors^13^. Control and p31^comet^-depleted cells were incubated with a sublethal dose (10 nM) of paclitaxel and imaged over a 72 h time course. We found that mitosis lasted 155.00 ± 199.86 min in paclitaxel-treated (n = 30), and 150.40 ± 295.99 min in p31^comet^-depleted (n = 30) cells, more than four times longer than control siRNA-cells (31.33 ± 4.34 min, n = 30) (Fig. 3c). Notably, combination of p31^comet^-depletion + paclitaxel resulted in a dramatic increase in mitosis duration (443.67 ± 365.92 min, n = 30), compared to control and to individual treatments (Fig. 3c), again suggesting that p31^comet^ downregulation, by preventing SAC silencing, delays mitotic slippage and retains low dose paclitaxel-treated cells in mitosis.

**Figure 4:**
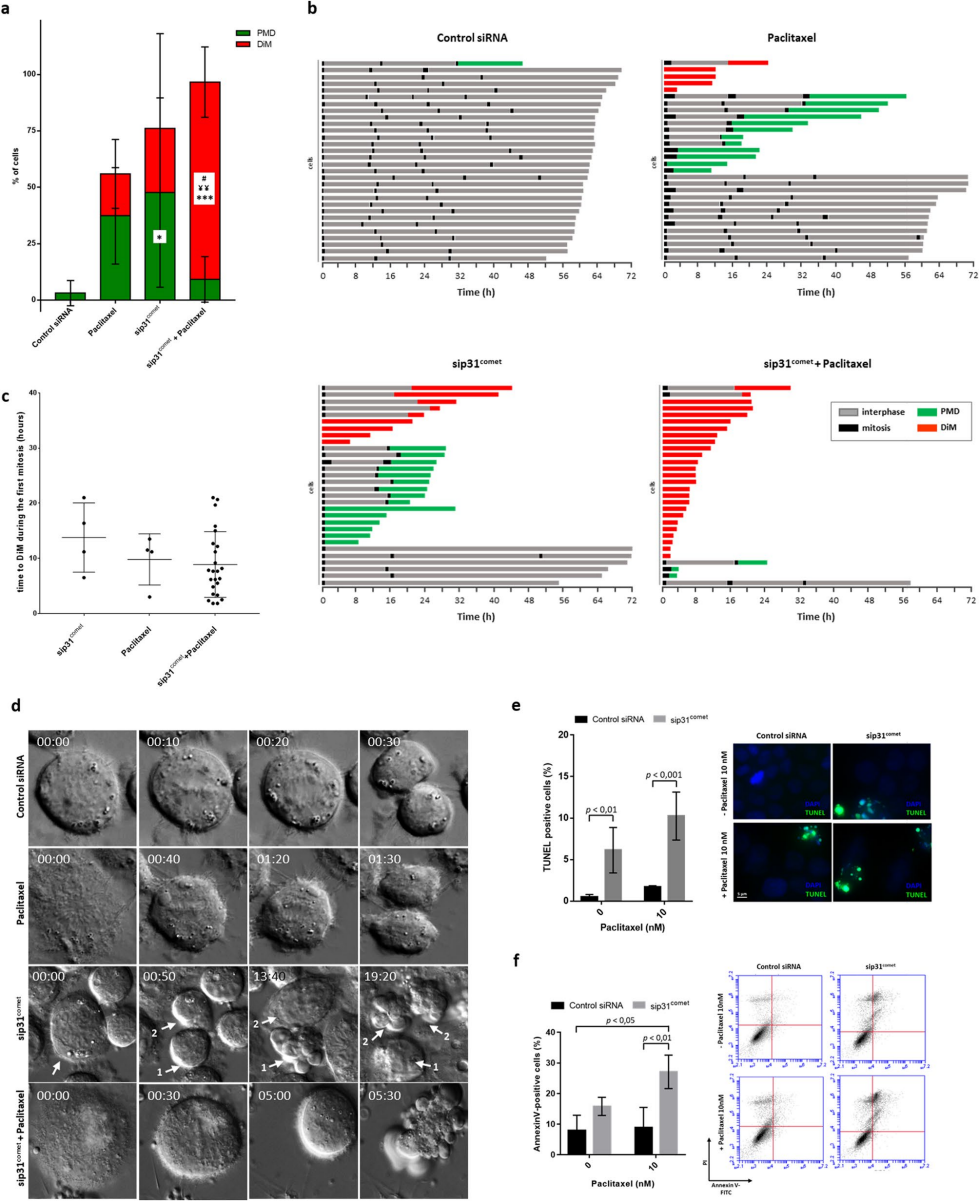
p31^comet^ knockdown enhances cell death in mitosis following addition of nanomolar doses of paclitaxel. (**a**) Quantification of the percentage of cells undergoing post-mitotic death (green) and death in mitosis (red) over the total number of cells. Cells were transfected with control or p31^comet^ siRNAs for 48 h, then paclitaxel was added for 24 h, and cells imaged by time-lapse microscopy for 72 h. *p < 0.05, ***p < 0.001, sip31^comet^ or sip31^comet^ + paclitaxel vs Control siRNA; ^#^*p* < 0.05, sip31^comet^ + paclitaxel *vs* sip31^comet^, ^¥¥^*p* < 0.01, sip31^comet^ + paclitaxel vs paclitaxel, by two-way ANOVA with Tukey's multiple comparisons test. *PMD* post-mitotic death, *DiM* death in mitosis. (**b**) Cell fate profiles, as determined by time-lapse microscopy. Cells were treated as in (**a**). The graphics represent the tracking from the time when cells entered mitosis (zero h). Individual cells are represented as horizontal bars. After mitosis, the time of cell death was determined by the time the first daughter cell dies. Thirty cells are represented per condition. (**c**) The scatter plot demonstrates the quantification of time from mitotic entry to death for cells which died during the first mitosis. Each spot represents one cell. (**d**) The panel shows time-lapse sequences representative of the cells characterized in (**b**). Paclitaxel and sip31^comet^-treated cells have longer mitosis than that treated with Control siRNA, which spend only 30 min in mitosis. A sip31^comet^-transfected cell (arrow) dies through PMD, and an increase of only 20 min in mitosis duration relatively to the Control siRNA-treated cell is enough to trigger death (1). One of the daughter-cell survives and divides (2), but her daughter cells die after mitosis. A cell treated with sip31^comet^ plus paclitaxel is trapped in mitosis and undergoes membrane blebbing after 5 h. (**e**) Cell death by apoptosis was tested by TUNEL assay to detect DNA fragmentation. Representative images are shown (left). DNA (blue) was stained with DAPI. DNA fragmentation is represented as green. Quantification of TUNEL positive is shown (right). (**f**) Flow cytometry analysis of apoptosis by Annexin V/PI co-staining, 48 h after paclitaxel treatment. Quantification of Annexin V-positive cells (left) and representative cytogram (right) are shown. The quadrants Q were defined as Q1 = live (Annexin V- and PI-negative), Q2 = early stage of apoptosis (Annexin V-positive/PI-negative), Q3 = late stage of apoptosis (Annexin V- and PI-positive) and Q4 = necrosis (Annexin V-negative/PI-positive). *p < 0.05, **p < 0.01, ***p < 0.001, by two-way ANOVA with Tukey's multiple comparisons test. For all the experiments, the error bars represent mean ± SD.

Cell survival profiling showed that the lethality of paclitaxel was increased after p31^comet^-depletion (Fig. 4a–c, and Supplementary Videos S1, S2, S3 and S4). 10 nM paclitaxel resulted in only 56.67% cell death. At this low concentration, while only a small fraction underwent DiM (16.67%) or PMD (40.00%) after one to three cycles, the remainder (43.33%) continued cycling. After p31^comet^-depletion, while 13.33% underwent DiM at the first mitosis, 86.67% divided. Of the dividers, 19.23% underwent DiM and 57.69% underwent PMD only after the first or second mitosis, while the survivors (23.08%) remained at interphase suggesting cell cycle arrest. Notably, combining paclitaxel with p31^comet^ depletion shifted the fate to DiM during the first (80%) mitosis and, interestingly, time to death was accelerated by 5.94 h and by 0,92 h comparatively to that of DiM in p31^comet^-depleted cells and paclitaxel-treated cells, respectively (Fig. 4d). In the few PMD events that occurred, time to death was significantly shortened, comparatively to individual treatments. Apoptosis was the main mechanism of cell death as confirmed by TUNEL assay and Annexin-V/propidium iodide costaining (Fig. 4e,f).

Overall, the results indicate that suppression of p31^comet^ prevents SAC silencing and delays mitotic slippage, thereby enhancing and accelerating cell death during the first mitosis, at clinically relevant doses of paclitaxel. Because the effect of the combination is close to the sum of the single effects, we conclude that the combined treatment has an additive effect.

### p31^comet^-siRNA mediated cell death can be accelerated by a BH3-mimetic drug

Variations in cell death sensitivity to antimitotics was attributed to two competitive and mutually exclusive networks, one controlling mitotic cell death through accumulation of apoptotic signals, and the other controlling mitotic slippage through gradual cyclin B1 degradation^22^. Thus, one way to force mitosis-arrested cells to die, rather than to slip, is to challenge them with small molecules that artificially stimulate apoptosis. We thought that by delaying premature SAC silencing and, simultaneously, stimulating apoptosis signal accumulation, one should create maximal conditions for maximal cytotoxicity. We explored this possibility by combining p31^comet^ knockdown with the BH3-mimetic drug Navitoclax, an antagonist of the Bcl-2 family of antiapoptotic proteins Bcl-2, Bcl-XL, and Bcl-w^23^. Mitotic duration and cell fate were analyzed by live-time imaging, over 72 h experiments, as above.

**Figure 5:**
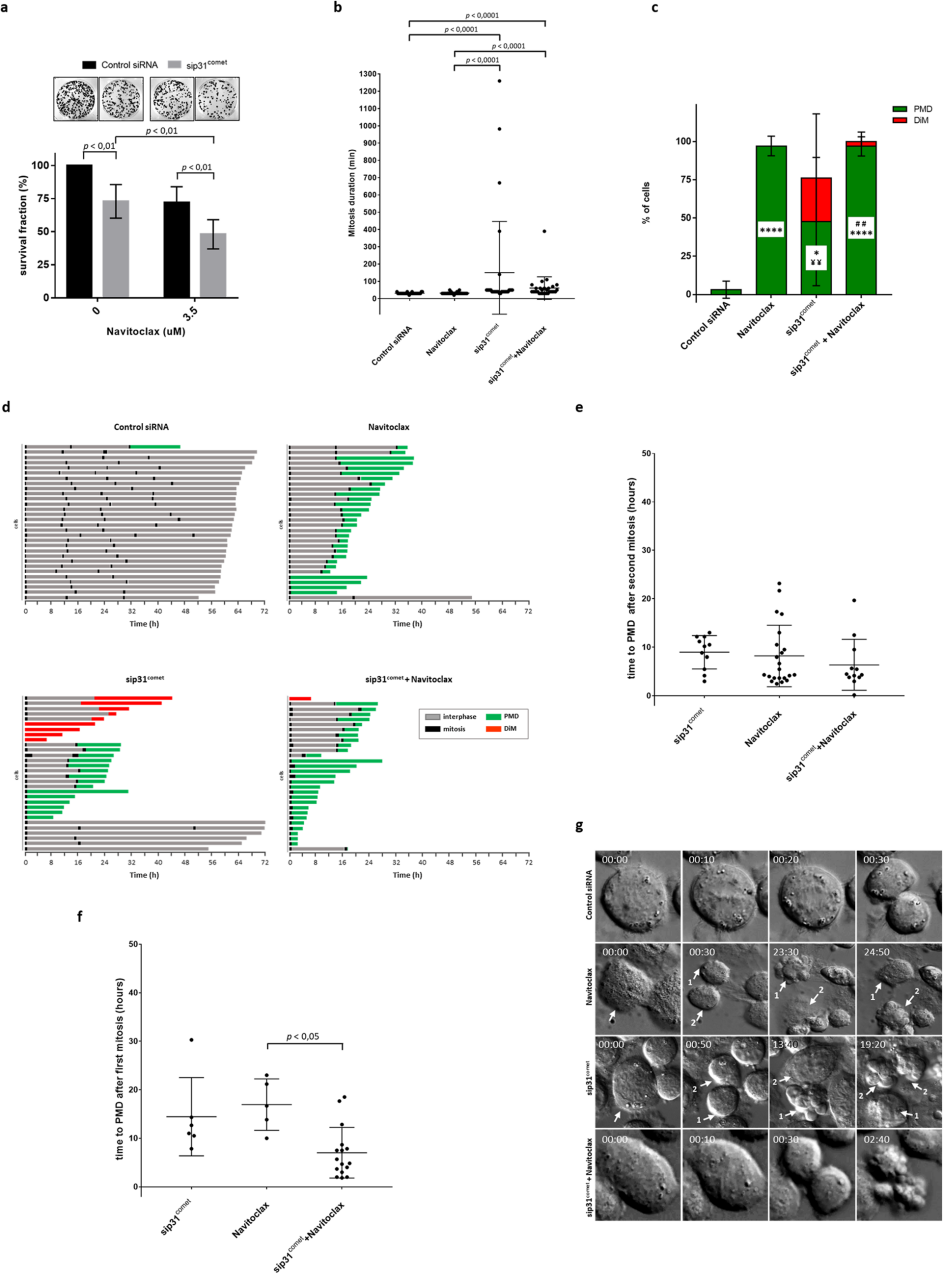
Navitoclax decreases mitosis duration and accelerates post-mitotic death in sip31^comet^-treated NCI-H460 cells. (**a**) Colony formation assays. 24 h post-transfection with control or p31^comet^ siRNAs, Navitoclax was added at the indicated concentrations and cells were incubated for an extra 48 h. Cells were then washed and allowed to grow for 10 days. Results are the mean from three independent experiments, expressed as % of survival fraction. Statistical analysis was performed by two-way ANOVA with Tukey's multiple comparisons test. (**b**) Mitosis duration as determined by live-cell imaging. NCI-H460 cells were treated with control or p31^comet^ siRNA, either alone, or in combination with 3.5 µM of Navitoclax. For the combined treatment, Navitoclax was added 24 h after siRNA transfection, and cells imaged for 72 h. The scatter plot shows the time from mitosis entry to cell division. Each spot represents one mitotic cell. Krushkal–Wallis with Dunn’s multiple correction test was used for statistical analysis. (**c**) Quantification of the percentage of cells undergoing post-mitotic death (PMD) and death in mitosis (DiM) over the total number of cells. Cells were treated as in (**b**). *p < 0.05, **p < 0.01, ***p < 0.001, Navitoclax or sip31^comet^ or sip31^comet^ + Navitoclax vs Control siRNA; ^##^*p* < 0.01, sip31^comet^ + Navitoclax vs sip31^comet^; ^¥¥^*p* < 0.01, Navitoclax vs sip31^comet^, by two-way ANOVA with Tukey's multiple comparisons test. (**d**) Cell fate profiles, as determined by time-lapse microscopy. Individual cells were tracked and represented as horizontal bars. The graphic represents the tracking from the time when cells entered in mitosis (zero h). After mitosis, the time of cell death is determined by the time the first daughter cell dies. Thirty cells are represented per condition. (**e**) The scatter plot shows the time from mitotic exit to death for cells which died after the second mitosis. Each spot represents one cell. Statistical analysis was performed Krushkal–Wallis with Dunn’s multiple correction test. (**f**) The same analysis as in (**e**), but for cells which died after the first mitosis. For all the experiments, the error bars represent mean ± SD. (**g**) The panel shows time-lapse sequences representative of the cells characterized in (**d**). A Navitoclax-treated cell spends the same time in mitosis as the Control siRNA-treated cell (30 min), and the first daughter cell (1) dies 23 h after division. The cell treated with sip31^comet^ plus navitoclax spends only 30 min in mitosis (20 min less than the cell only transfected with sip31^comet^), and both the daughter-cells die approximately 2 h after cell division, 21 h or 11 h less than cells individually treated with Navitoclax or sip31^comet^, respectively.

First, we observed that addition of Navitoclax further compromised long-term survival of cells depleted of p31^comet^ (Fig. 5a). As shown in Fig. 5b, exposure to 3.5 µM Navitoclax alone did not alter mitosis duration in control siRNA cells. Interestingly, addition of Navitoclax to p31^comet^ siRNA transfected cells significantly reduced the duration of the mitotic block to 61.00 ± 65.51 min (n = 30), more than two times shorter compared to p31^comet^ siRNA transfected only cells (150.40 ± 295.99 min (n = 30). Because no mitotic role was described, so far, for the antiapototic proteins targeted by Navitoclax, we believe that the shortening of the observed mitotic arrest time is the result of precocious cell death, rather than a genuine reduction in mitotic arrest duration.

As to cell survival profile (Fig. 5c,d), Navitoclax alone induced PMD in 96.67% of treated cells, most of which (86.21%) occurred only after the second cell cycle, with an average of 7.81 h between mitotic exit and death (Fig. 5e). When Navitoclax was added to p31^comet^-depleted cells, PMD after the first mitosis became the predominate cell fate (56.67%) (Fig. 5d), with death onset time significantly accelerated by approximately 8 h and 4 h, compared to Navitoclax (*p* < 0.05) and p31^comet^-depletion individual treatments, respectively (Fig. 5f,g, and Supplementary Videos S5 and S6). Interestingly, although some PMD (41.38%) still occurred only after the second cell cycle (Fig. 5c,d), the death onset time was significantly accelerated by approximately 1.19 h relatively to Navitoclax alone, and 5.06 h relatively to p31^comet^-depletion alone (Fig. 5f), indicating that cells that escape cell death after the first cell cycle are committed to death after the second cell cycle.

In sum, the data demonstrate that the use of a BH3-mimetic in an antagonized SAC silencing background enhances and accelerates cancer cell death, largely by post-mitotic cell death after the first division.

## Discussion

In this study we demonstrated that modulating SAC silencing, by p31^comet^ depletion, can influence mitotic slippage and cell death in the presence of spindle poisons or apoptosis potentiators. While both spindle poisons and apoptosis potentiators exacerbate cell death in p31^comet^-depleted cells, they behave differently with regard to their mechanism. Depletion of p31^comet^ extends the duration of mitotic block in the presence of paclitaxel, and shifts cell fate to accelerated cell death in the first mitosis. In contrast, p31^comet^ depletion shifts cell fate to accelerated post-mitotic death after the first cell cycle, in the presence of the apoptotic potentiator Navitoclax. Thus, in both contexts, cell death is enhanced and accelerated comparatively to individual treatment. We also show that p31^comet^ is overexpressed in NSCLC cell lines. Our data highlight the relevance of p31^comet^ as drug target for cancer therapy.

We demonstrated that that p31^comet^ targeting enhances cancer cells exposed clinically relevant doses of paclitaxel, in an additive manner. Upon p31^comet^ depletion plus paclitaxel, cells were trapped in mitosis and arrested until death. This is in concordance with a previous work showing that p31^comet^ depletion promoted an increased duration of mitosis in the presence of paclitaxel that culminated with cell death in a colorectal carcinoma cell line^2^. However, this result was achieved under a higher concentration of paclitaxel than that used in our study. Paclitaxel inhibits tubulin depolymerization, impairing microtubule dynamics and leading to the permanent activation of SAC^3^. However, SAC can be satisfied under low concentrations of paclitaxel, allowing mitosis to proceed in the presence of spindle abnormalities and congregation errors, which may increase the aggressiveness of malignant cells^24,25^. Thus, by delaying SAC silencing, p31^comet^ knockdown enhances and accelerates cell death in the presence of low concentrations of paclitaxel, most probably by delaying mitotic slippage which favors accumulation of apoptotic signals. Noteworthy, the precise mechanism of paclitaxel cytotoxicity is still debatable. In addition to its antimitotic-mediated chemotherapeutic effect, paclitaxel was shown to trigger proinflammatory response by activation of innate immunity, providing an opportunity to explore its combination with immune checkpoint inhibitors^26^. Interestingly, rather than inducing mitotic delay, low doses paclitaxel promote chromosome missegregation and micronucleation which stimulates innate immunity response and promotes antitumor immune surveillance^27^.

We found that addition of the apoptosis potentiator Navitoclax to p31^comet^ depleted cells accelerated post-mitotic death. Our results are in line with previous reports demonstrating that Navitoclax plus antimitotics co-treatment potentiated cancer cell death^28,29^. Surprisingly, although some studies have shown that Navitoclax in combination with MTAs prompted mitotic death by accelerating apoptosis in the mitotic-arrested cells^28,29^, we demonstrated that Navitoclax shifted cell death from DiM to PMD in p31^comet^ depleted cells. This result is in line with another study that showed that inhibition of Bcl-xL by the BH3-mimetic WEHI-539 induces PMD in RKO cells in the presence of paclitaxel^24^. We also found that Navitoclax promotes post-mitotic death of p31^comet^-depleted cells after a short delay in mitosis. This suggests that cells that exit mitosis after a delay, here caused by p31^comet^ knockdown, are committed to die due to Navitoclax-mediated inhibition of antiapototic proteins. Indeed, Bcl-xL (a Navitoclax target) was shown to be crucial for cell survival following an abnormal mitosis^24^. Intriguingly, Navitoclax treatment alone resulted in a strong cytotoxic response (Fig. 5c). However, Navitoclax showed limited single-agent activity in a completed phase II study, and current studies are focusing on combination therapies^30,31^. In this perspective, we believe that its use to enhance/accelerate apoptotic signal accumulation in cancer cells treated with antimitotics could be beneficial by avoiding slippage from mitotic arrest. The p31^comet^ knockdown/Navitoclax combination could provide maximal conditions for maximal cytotoxicity. Indeed, mechanistically, the combination is much more aggressive in that it promotes rapid cell death, already at the first mitosis, while most of cell killing occurred at mitosis of the second cell cycle in individual treatments (Fig. 5d). This is corroborated by the enhanced reduction of cell survival (Fig. 5a), and could be clinically relevant as it may offer higher efficacy while reducing repeated administration.

p31^comet^ overexpression was previously associated with the abolishment of SAC-dependent mitotic arrest and subsequent mitotic slippage^2,32,33^, as well as with an increased resistance to apoptosis and to antimitotic drugs, such as paclitaxel in cancer cells^32^. Furthermore, mRNA screenings published in oncomine database (www.oncomine.org) suggested that p31^comet^ is overexpressed in several cases of lung cancer as well as in other cancers. In line with those evidences, we confirmed that p31^comet^ was overexpressed at mRNA and protein level in three NSCLC cell lines when compared with a non-tumoral cell line, thus highlighting its potential value as a target for NSCLC therapy.

In conclusion, our data suggest that targeting SAC silencing components, such as p31^comet^, can provide a mean to block mitosis and, at the same time, to delay mitotic slippage, thereby providing maximal conditions to enhance cytotoxicity of microtubule poisons and apoptosis-promoting agents. Therefore, targeting SAC silencing can provide a rationale for combination chemotherapy against cancer that deserves to be further explored in a goal to overcome problems of resistance and side effects.

## Materials and methods

### Cell lines and culture conditions

Cells were grown and maintained as described^34^. NCI-H460 (human non-small cell lung cancer) cells were grown in RPMI-1640 culture medium (Lonza, Basel, Switzerland) with 5% FBS. HPAEpiC (human pulmonary alveolar epithelial cells), A549 (human adenocarcinoma alveolar basal epithelial), and Calu-3 (human lung adenocarcinoma) cells were grown in DMEM medium with 10% fetal bovine serum (FBS, Biochrom) and 1% non-essential amino acids (Sigma Aldrich Co., Saint Louis, MO, USA). Cells were maintained in a 5% CO_2_ humidified incubator, at 37 °C. The experiments were performed when cells were at exponentially growing and presented more than 95% viability. The NCI-H460, A549, and Calu-3 cell lines were obtained from American Type Culture Collection. HPAEpiC cell line was purchased from ScienCell Research Laboratories.

### RNA isolation and quantitative real-time PCR

RNA isolation for quantitative real-time PCR was performed using the PureZOL RNA Isolation Reagent (Bio-Rad Laboratories, Hercules, CA, USA), according to the manufacturer's instructions. Quantification of RNA was achieved by spectrophotometry (NanoDrop 2000, Thermo Scientific, Waltham, MA, USA). cDNA was synthesized with the iScript cDNA Synthesis Kit (Bio-Rad), according to supplier’s instructions. The iQ SYBR Green Supermix Kit (Bio-Rad) was used for amplification on iQ Thermal Cycler (Bio-Rad) coupled to CFX Manager Software (version 3.1, Bio-Rad), as follows: initial denaturing step at 95.0 °C for 3 min; 40 cycles at 94.0 °C for 20 s; 65.0 °C for 30 s and 72.0 °C for 30 s. Temperatures from 65.0 to 95.0 °C, with increments of 0.5 °C for 5 s were included in the melt curves. Primers were used at final concentration of 0.1 µM. The following primer sequences were employed: p31^comet^, 5′-AGTCCCTGATTTGGAGTGGT-3′ (forward), 5′-GTAAACTGACAGCAGCCTTCC-3′ (reverse); actin, 5′-AATCTGGCACCACACCTTCTA-3′ (forward), 5′-ATAGCACAGCCTGGATAGCAA-3′ (reverse); GAPDH, 5′-ACAGTCAGCCGCATCTTC-3′ (forward), 5′-GCCCAATACGACCAAATCC-3′. For each data point, triplicated experiments were performed. The results were normalized against GAPDH and actin and expression levels and analyzed through the ΔCT method. Over- or underexpression of a gene was determined with basis on a fold value of mRNA level ≥ or ≤ 1.5 relatively to that of normal cells.

### siRNAs transfection

NCI-H460 cells were seeded at density of 0.1275 × 10^6^ cells per well, in 6-well plates containing complete culture medium. Cells were transfected after 24 h using INTERFERin siRNA Transfection Reagent (PolyPlus, New York, USA) following the manufacter’s instructions. Transfection was performed with 50 nM of a validated siRNA sequence against p31^comet^^18^ or a validated negative control siRNA (AllStars Negative Control siRNA, Qiagen, Germantown MD, USA).

### Cell extracts and Western blotting

Preparation of total cell protein extracts was performed as previously described^35^. For Western Blot analysis, samples were separated by molecular weight using SDS-PAGE gels and transferred to a nitrocellulose membrane. The membrane was blocked with 0.05% Tween-20 with 5% w/v nonfat dry milk and the incubation with antibodies was performed within the same solution. The signal was detected using ECL detection of the HRP-conjugated secondary antibodies. Blots were visualized using X-ray films. Images of X-ray films were captured using Carestream BIOMAX Light Film (Sigma-Aldrich) and quantified by densitometry using ImageJ 1.4v software (http://rsb.info.nih.gov/ij/). The primary antibodies were used as follow: rabbit anti-p31^comet^ (abcam) and mouse anti-α-tubulin (Sigma-Aldrich), diluted at 1/1000 and 1:5000, respectively. Horseradish peroxidase (HRP)-conjugated secondary antibodies were diluted at 1:4000 (anti-mouse, Sigma-Aldrich) or at 1:1000 (anti-rabbit, Sigma-Aldrich). ImageJ 1.4v software was used for the quantification of the intensity of the protein signal. α-Tubulin expression levels were used for normalization.

### Mitotic index determination

NCI-H460 cells were seeded in 6-well plates containing complete culture medium at density of 0.1275 × 10^6^ cells per well. Cells were counted 48 h after transfection with control- or p31^comet^ siRNA, or 24 h after paclitaxel treatment. For the p31^comet^ siRNA and paclitaxel cotreatment, paclitaxel was added 24 h after siRNA transfection. Cells were counted from random microscope fields (n > 2000 for each condition). Round-shaped mitotic cells were quantified over the total cell population for the determination of the mitotic index. Paclitaxel (Sigma-Aldrich) was used at a clinically relevant concentration of 10 nM.

### Cell viability assay

The MTT (3-(4,5-dimethylthiazolyl-2)-2,5-diphenyltetrazolium bromide) assay (Sigma-Aldrich) was used to determine cell viability. Control and p31^comet^ siRNA-treated cells were seeded in 96-well plate at the density of 5000 cells per well. After 6 h, paclitaxel was added at a clinically relevant range of concentrations (0–100 nM)^25,36^. 48 h later, culture medium was replaced with fresh FBS-free medium containing 20 µl of MTT reagent (5 mg/ml in PBS). Cells were at 37 °C and 5% CO_2_ for 4 h. Solubilization of the purple formazan crystals was achieved with a detergent solution (89% (v/v) 2-Propanol, 10% (v/v) Triton X-100, 1% (v/v) HCl 3.7%), for 2 h. Results were analyzed with basis on optical density at 570 nm. Measurements were performed in a microplate reader (Biotek Synergy 2, Winooski, VT, USA) coupled with the Gen5 software (version 1.07.5, Biotek, Winooski, VT, USA). Cell viability values were normalized against control siRNA-treated cells.

### Colony forming assay

Colony formation assays were performed as described^34^. 24 h after control- or p31^comet^ siRNA-transfection, NCI-H460 cells were seeded in six-well plates at the density of 500 cells per well. Cells were allowed to attach for 6 h and then treated with paclitaxel (2 and 4 nM and 10 nM) and/or Navitoclax (3.5 µM). 48 h later, the medium was removed, cells were washed gently with PBS, and fresh medium was added. Cells were allowed to grow for 10 days, and the recovered colonies were fixed with 3.7% (w/v) paraformaldehyde in PBS for 5 min and stained with 0.05% (w/v) violet crystal (Merck Millipore, Billerica, MA, USA) in distilled water for 20 min. Three independent experiments were performed on duplicate dishes for each condition. The number of colonies was counted and the plating efficiency (PE) was calculated as the percentage of the number of colonies over the number of cells seeded in control. The survival fraction (the number of colonies over the number of cells seeded × 1/PE) was determined for each condition.

### Flow cytometry

NCI-H460 cells were plated in six-well plated at the density of 1,275,000 cells per well. 24 h after control- or p31^comet^ siRNA-transfection, cells were treated with 10 nM or 100 nM of paclitaxel. 24 or 48 h later, cells were harvested and prepared for flow cytometry, as previously described^34^. Cell cycle analysis was performed after treatment with propidium iodide and RNase. Briefly, cells were harvested, washed twice in PBS, fixed in 70% ice-cold ethanol and maintained at 4 °C for at least 30 min. Cells were then treated with 5 µg/ml propidium iodide and 100 µg/ml RNase in PBS for 30 min and analyzed in the flow cytometer. Apoptosis detection was performed with the Annexin V-FITC Apoptosis Detection Kit (eBioscience, Vienna, Austria) according to manufacturer's instructions. Flow cytometry analysis was carried out using a BD Accuri C6 Flow cytometer (BD Biosciences, Qume Drive, San Jose, CA) with the analysis of 20,000 events per sample. Data was analyzed with BD Accuri C6 Plus Software, version 1.0.27.1 (www.bdbiosciences.com).

### TUNEL assay

In order to detect DNA breaks, Terminal deoxynucleotidyl transferase-mediated nick end labeling (TUNEL) assay was performed. Briefly, cells were plated and treated as above in six-well plates containing coverslips. Coverslips-attached cells were processed with DeadEnd Fluorometric TUNEL System (Promega, Madison, WI, USA), according to the manufacturer's instructions. For DNA staining, 2 µg/ml of DAPI was used in Vectashield mounting medium. TUNEL-positive cells were scored in a total of 500 cells, from at least ten random microscopic fields, under fluorescence microscope, in order to determine the levels of cells undergoing cell death.

### Live cell imaging

For live-cell imaging experiments, 5.5 × 10^6^ NCI-H460 cells were seeded onto LabTek II chambered cover glass (Nunc, Penfield, NY, USA). Cells were allowed to attach for 24 h at 37 °C with 5% CO_2_, and then transfected with control or p31^comet^ siRNA or treated with 10 nM of paclitaxel, or 3.5 µM of Navitoclax. For cotreatment, paclitaxel or Navitoclax were added 24 h after siRNA-transfection. Time-lapse imaging was performed 24 h after siRNA transfection or immediately after the addition of paclitaxel or Navitoclax. RPMI without phenol red supplemented with 5% FBS was used in the experiments. Image capture was performed up to 72 h at intervals of 10 min under differential interference contrast (DIC) optics, with a 63× objective. An Axio Observer Z.1 SD inverted microscope (Carl Zeiss, Germany) coupled with an incubation chamber with the temperature set to 37 °C and an atmosphere of 5% CO_2_ was used in the experiments. ImageJ software (version 1.44, Rasband, W.S., ImageJ, US National Institutes of Health, Bethesda, MD, USA) was used to produce movies from the images captured during time-lapse imaging. The cells were followed through the entire imaging period and cell fates were tracked for each experimental condition. The number of cells were scored for mitosis and cell death with basis on changes in cell morphology by DIC imaging. Cell death was characterized by cell retraction and plasma membrane blebbing, and mitotic entry by cell rounding. Dead cells were categorized into death in mitosis (DiM) or post-mitotic death (PMD) when death occurred before or following cell division, respectively.

### Microscopy analysis and image processing

A Nikon TE 2000-U microscope (Amsterdam, Netherlands), with a DXM1200F digital camera (Amsterdam, Netherlands) and a Nikon ACT-1 software (version 2.62, Melville, NY, USA) was used for phase-contrast experiments, as described previously^34^. The acquisition of fluorescence images was performed in a Plan Apochromatic 63x/NA 1.4 objective on an Axio Observer Z.1 SD microscope (Carl Zeiss, Germany), coupled to an AxioCam MR3. For Z-stacks generation, the AxioVision Release SPC software (version 4.8.2, Carl Zeiss, Germany), with 0.4 mm intervals and after image deconvolution was used. ImageJ (version 1.44, Rasband, W.S., ImageJ, US National Institutes of Health, Bethesda, MD, USA) was used for image processing.

### Statistical analysis

Data were collected in the same experiments, being control siRNA and p31^comet^-siRNA only conditions the same for all the data. For statistical analysis, Unpaired Student t-test or ordinary two-way ANOVA with Tukey’s multiple comparisons test, or Krushkal–Wallis with Dunn’s multiple correction test were performed in GraphPad Prism version 7 (GraphPad software Inc., CA, USA). Data are shown as the means ± standard deviation (SD) of at least three independent experiments.

## Supplementary Information

Supplementary Information 1.

Supplementary Video S1.

Supplementary Video S2.

Supplementary Video S3.

Supplementary Video S4.

Supplementary Video S5.

Supplementary Video S6.
