# Supplementary material for: Antagonizing the spindle assembly checkpoint silencing enhances paclitaxel and Navitoclax-mediated apoptosis with distinct mechanistic
Source: Sci Rep. 2021 Feb 18;11:4139. doi: 10.1038/s41598-021-83743-7 (PMC7893169; doi:10.1038/s41598-021-83743-7)
Supplement: Supplementary file 1 — Supplementary Information 1. [file 41598_2021_83743_MOESM1_ESM.docx]

**Antagonizing the spindle assembly checkpoint silencing enhances paclitaxel and Navitoclax-mediated apoptosis with distinct mechanistic**

Ana C. Henriques^a,b,c^, Patrícia M. A. Silva^a^, Bruno Sarmento^a,b,d^, Hassan Bousbaa^a,c^

^a^ CESPU, Instituto de Investigação e Formação Avançada em Ciências e Tecnologias da Saúde, Instituto Universitário de Ciências da Saúde, Gandra PRD, Portugal

^b^ INEB, Instituto Nacional de Engenharia Biomédica, Universidade Do Porto, Porto, Portugal

^c^ Centro Interdisciplinar de Investigação Marinha e Ambiental (CIIMAR/CIMAR), Universidade Do Porto, Porto, Portugal

^d^ i3S - Instituto de Investigação e Inovação Em Saúde, Universidade Do Porto, Porto, Portugal

*Corresponding author: Hassan Bousbaa, Instituto de Investigação e Formação Avançada em Ciências e Tecnologias da Saúde, CESPU, Rua Central da Gandra 1317, 4585-116 Gandra, Paredes, Portugal; Phone: +351-224-157-186; Fax: +351-224157102; Email: hassan.bousbaa@iucs.cespu.pt

**Supplementary Information**


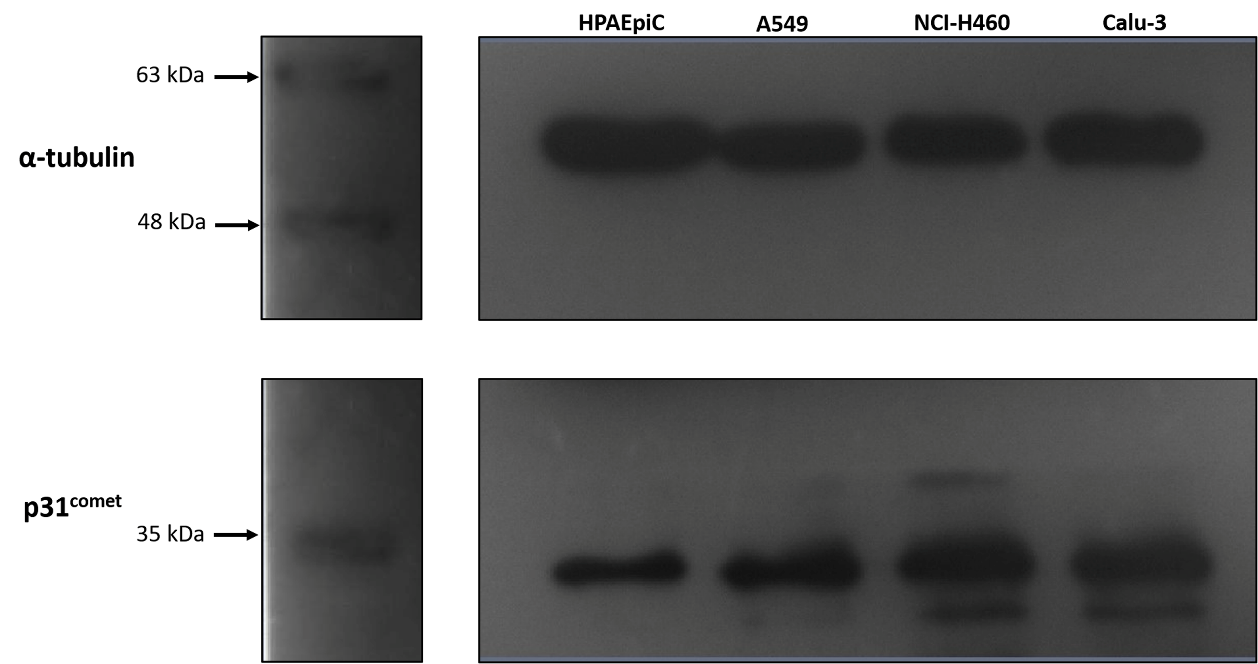


Supplementary Figure S1. Full length blots of p31^comet^ expression in HPAEpiC, A549, NCI-H460, and Calu-3 cell lines. The two blots are from the same membrane but were separated to avoid oversaturation of tubulin signal, because α-tubulin needed short exposure time, while p31^comet^ protein needed long exposure time.


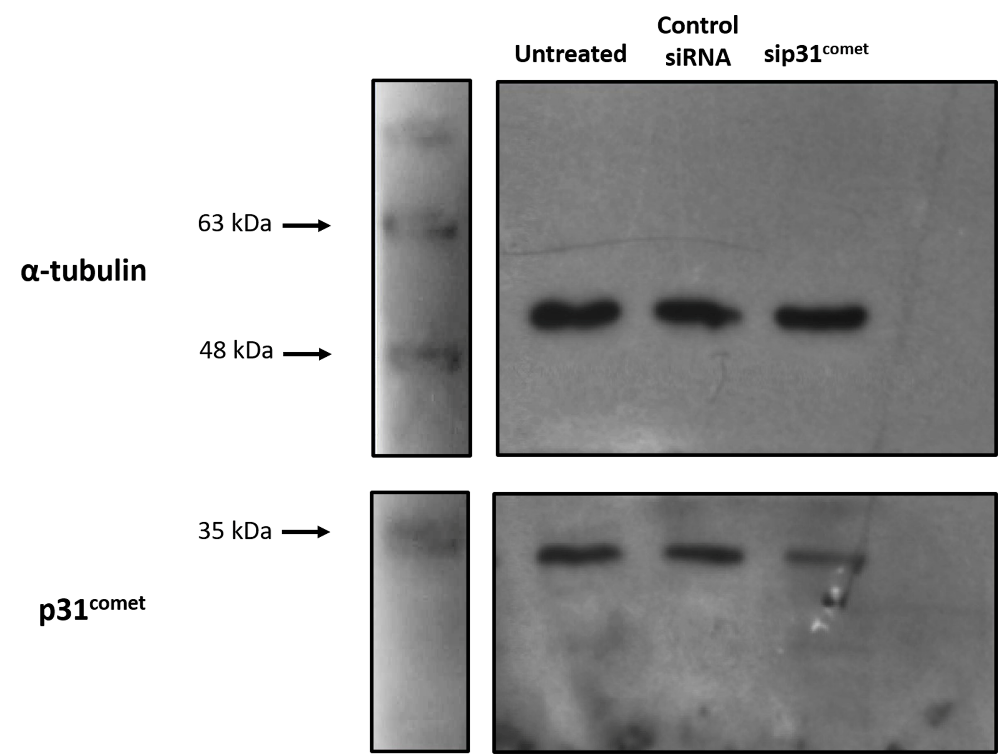


Supplementary Figure S2. Full length blots of p31^comet^ siRNA-mediated knockdown in NCI-H460 cells. The two blots are from the same membrane but were separated to avoid oversaturation of tubulin signal, because α-tubulin needed short exposure time, while p31^comet^ protein needed long exposure time.

Supplementary Video S1. Time-lapse sequence from Control siRNA transfected NCI-H460 cells.

Supplementary Video S2. Time-lapse sequence from NCI-H460 cells treated with paclitaxel (10nM).

Supplementary Video S3. Time-lapse sequence from p31^comet^ siRNA transfected NCI-H460 cells.

Supplementary Video S4. Time-lapse sequence from NCI-H460 cells treated with p31^comet^ siRNA and paclitaxel (10nM).

Supplementary Video S5. Time-lapse sequence from NCI-H460 cells treated with Navitoclax (3.5 µM).

Supplementary Video S6. Time-lapse sequence from NCI-H460 ells treated with p31^comet^ siRNA and Navitoclax (3.5 µM).
